# Immunomodulatory potential of black soldier fly larvae: applications beyond nutrition in animal feeding programs

**DOI:** 10.1093/tas/txac084

**Published:** 2022-06-22

**Authors:** Elizabeth Koutsos, Bree Modica, Tarra Freel

**Affiliations:** EnviroFlight, LLC, 1118 Progress Way, Maysville, KY 41056, USA; EnviroFlight, LLC, 1118 Progress Way, Maysville, KY 41056, USA; EnviroFlight, LLC, 1118 Progress Way, Maysville, KY 41056, USA

**Keywords:** antimicrobial peptides, black soldier fly, chitin, immunomodulatory, lauric acid, nutrition

## Abstract

Insect-derived ingredients, including whole larvae, protein-rich meal, and oil, have been extensively studied in recent years and shown to be a sustainable source of quality nutrition for virtually all animal species and life stages. In addition to the ability to use these ingredients as a source of essential nutrition, more recent research has demonstrated the potential for the immunomodulatory activity of various components of insect-derived ingredients. For all insects studied, antimicrobial peptides make up a critical part of the insects’ innate immune system and these peptides have antimicrobial efficacy when purified from hemolymph and tested in vitro. From black soldier fly larvae, in particular, lauric acid is a predominant fatty acid deposited into the insect, and lauric acid also has potential antimicrobial activity in vitro and in vivo. Finally, the chitin and chitosan components of the insect exoskeleton may modulate microbial activity in a variety of ways. In companion animals, poultry, and livestock species, insect-derived ingredients have shown the potential to reduce the impact of actual or simulated disease challenge on several parameters of animal health and well-being. This review describes the current state of knowledge of the immunomodulatory potential of insect-derived ingredients.

## INTRODUCTION

The black soldier fly (*Hermetia illucens*) is a true fly (Diptera) of the family Stratiomyidae, native to South America, but has now spread to many other tropical and warmer temperate regions of the world (James, 1935; Callan, 1974; Kaya et al., 2021). The adult fly resembles an all-black wasp in appearance and is 13 to 20mm in length (May, 1961; Tomberlin and Sheppard, 2001). Adults lack mouthparts and therefore do not feed or transmit disease (Newton et al., 2005). The larvae are whitish in color, up to 20 mm in length, 6 mm in width and weigh up to 220 mg when mature (Hall and Gerhardt, 2002). The larvae feed on various decaying organic materials (e.g., fruits and vegetables, distillers’ grains, animal manure), reducing the volume of organic matter by 42% to 56% by incorporating protein into their bodies at as much as 40+% protein and 30+% fat (Newton et al., 2005). Black soldier flies also reduce harmful bacteria and housefly presence (Sheppard, 1983). Due to the capacity to upcycle low-value feed inputs, and diverting those inputs from a landfill or other environmentally detrimental outcomes, the black soldier fly larvae (BSFL) have received significant interest in recent years. This review will focus on the benefits of BSFL beyond providing nutrition.

## BSFL-DERIVED INGREDIENTS AS A SOURCE OF ESSENTIAL NUTRITION

Insects can be a source of essential nutrition for many animal species, and for many species of farmed and companion animals, insects are part of their wild-type diets. High quality and sustainable nutrient output, coupled with high feed conversion efficiency, make insects ideal for commercial production. Some current commercially produced insects include mealworms (*Tenebrio molitor*), crickets (*Acheta domesticus*), and the black soldier fly larvae (*Hermetia illucens*). Compared to some traditional agriculture species, black soldier fly larvae can produce more than 3,000 times as much protein per acre with significantly less resource inputs in the forms of feed, water, and land (Koutsos et al., 2019). Additionally, the use of insects reduces CO_2_ production and fossil resource depletion compared to some other protein production systems, such as wild-caught fishmeal (Van PhI et al., 2020). Black soldier fly larvae can be fed as a whole (dried or live) larvae or as BSFL-derived ingredients, such as BSFL meal (ground, partially defatted larvae) and BSFL oil (oil extracted from the whole insect). Additionally, frass (the leftover product from feeding larvae, including larvae waste, remaining feed ingredients, and exoskeleton sheddings) can be used as a feed ingredient or plant fertilizer. Other potential ingredients such as hydrolyzed protein, chitin, and other functional compounds may be produced by further processing. For all insect-derived ingredients, it is important to keep in mind that composition and bioavailability can be altered by factors such as processing methods and feeding program (Koutsos et al., 2021). However, in general, research has shown that BSFL meal and oil can be an effective replacement for protein and energy sources, including soybean meal and oil, fishmeal, poultry by-product meal, and others for poultry, swine, fish, and companion animals (Widjastuti et al., 2014; Al-Qazzaz et al., 2016; Mwaniki et al., 2018; Secci et al., 2018; Dalle Zotte et al., 2019; Chia et al., 2021; Freel et al., 2021; Oteri et al., 2021). In the United States, BSFL (whole and partially defatted meal) is currently approved for feeding to poultry, salmonids, swine, and wild birds, with tentative approval for adult dogs as of the writing of this manuscript. BSFL oil is currently tentatively approved for feeding to finfish and swine.

## IMMUNOMODULATORY POTENTIAL OF BSFL-DERIVED INGREDIENTS

Numerous trials have shown the potential of BSFL meal and oil to provide protein and energy in animal diets. More recent work has examined the potential of these ingredients, and some fractionated components, to enhance or optimize immune function. In particular, three major components of BSFL ingredients are under investigation: antimicrobial peptides, lauric acid, and chitin.

### Antimicrobial Peptides

Insects lack adaptive immunity and instead rely on innate immunity, consisting of cellular and humoral defense systems (Park et al., 2014; Jo et al., 2017; Yang et al., 2018; Zdybicka-Barabas et al., 2017). The insect cellular immune system is meditated by hemocytes (structurally and functionally similar to mammalian neutrophils) that induce processes such as phagocytosis, and the humoral immune system includes protection via the phenoloxidase cascade and antimicrobial peptides (Lu et al., 2014; Yang et al., 2018). In comparison, the innate immunity of mammals includes a cellular system mediated by myeloid cells, and a humoral system that also includes antimicrobial peptides (Sheehan et al., 2018). Additionally, mammals have an adaptive immune system that provides specific responses to pathogens via lymphocytes with specific receptors, as well as provides prophylactic protection due to memory of previous infections (Sheehan et al., 2018). Although insects lack adaptive immunity, they do exhibit immunological priming that increases responses to infections previously encountered (Sheehan et al., 2018).

Antimicrobial peptides (AMPs), as a component of the insect immune system, inhibit the invasion of harmful pathogens to the insect host (Harlystiarini et al., 2019). They are synthesized in the insect fat body, analogous to the mammalian liver, and subsequently secreted in the hemolymph (Reichhart et al., 1992; Kang et al., 1998; Park et al., 2014). Currently, according to published research, a total of 57 genes coding for recognized AMPs have been identified in BSFL adults and larvae (Vogel et al., 2018; Moretta et al., 2020). Of these 57 identified genes, 53 have been identified from the larval stage with 26 coding for defensin AMPs; at the time of this review, this is the highest number reported for any invertebrate (Vogel et al., 2018). The only other insect with a similar number of identified AMP genes is the invasive harlequin ladybird (*Harmonia axyridis*) with 52 AMP genes identified (Vilcinskas et al., 2013). Other Dipteran species from which AMPs have been identified include the adult fruit fly (*Drosophila melanogaster*) (Carlsson et al., 1991), adult tsetse fly (*Glossina morsitans*) (Carlsson et al., 1991), adult fleshfly (*Sarcophaga peregrine*) (Leem et al., 1996), larvae of sawfly (*Acantholyda parki*) (Leem et al., 1996), larvae of grey fleshfly (*Neobellieria bullata*) (Meylaers et al., 2003), larvae of the housefly (*Musca domestica*) (Hou et al., 2007), and larvae of the common green bottle fly (*Lucilia sericata*) (Bexfield et al., 2008). Additionally, mealworms (*T*. *molitor)* have been found to exhibit AMP activity at the egg, larval, and adult stages (Dobson et al., 2012; Jacobs et al., 2017; Yang et al., 2018; El Shazely et al., 2019). Interestingly, BSFL are the first insect larvae to have any identified AMP activity against *Helicobacter pylori*. Alvarez et al. (2019) identified four AMPs with such activity from BSFL hemolymph. Although these four AMPs represented less than 10% of all the identified genes coding for AMPs, their activity was comparable to metronidazole, a common antibiotic used to treat *H*. *pylori* and other infections.

Using extracts of hemolymph, functional antimicrobial activity has been demonstrated in vitro against gram-negative bacteria (*Escherichia coli*, *Enterobacter aerogenes*, *Helicobacter pylori*, *Pseudomonas aeruginosa*, *Pseudomonas fluorescens, Salmonella* spp.), gram-positive bacteria (*Bacillus subtilis, Kocuria rhizophila*, *Mircrococcus luteus*, *Staphylococcus aureus*) and yeast (*Candida albicans*) (Choi et al., 2012; Park et al., 2014; Vogel et al., 2018; Alvarez et al., 2019; Harlystiarini et al., 2019; Table 1). Hemolymph extraction can employ sequential steps, the most common being: 1) acidic methanol extraction method to extract small molecules while denaturing and precipitating larger molecules and 2) chloroform and ethyl acetate extraction method to remove lipids (Park et al., 2014; Vogel et al., 2018). Methanol-extracted BSFL hemolymph has demonstrated greater antibacterial efficacy compared to chloroform-extracted hemolymph (Choi et al., 2012; Park et al., 2014; Harlystiarini et al., 2019). Inhibition of gram-negative bacteria (*E. coli*, *E*. *aerogenes*, and *P*. *aeruginosa*), gram-positive bacteria (methicillin-resistant *S*. *aureus*, *K*. *rhizophila*, *M*. *luteus*, *B*. *subtilis*), and yeast (*C*. *albicans*) by methanol-extracted hemolymph occurred at much lower minimum inhibitory concentrations (MIC) of either 12.5 or 25 mg/mL as compared to chloroform-extracted hemolymph (>100 mg/mL MIC) (Park et al., 2014). Methanol-extracted BSFL hemolymph was also more effective than positive controls (chloramphenicol and hypochlorous acid) when tested against biofilms of *E. coli*, *P*. *fluorescens*, *M*. *luteus*, and *B*. *subtilis* grown on microtiter plates (Müller et al., 2017). Additionally, Choi et al. (2012) demonstrated greater effectiveness after 12 h against *Klebsiella pneumoniae*, *Neisseria gonorrhoeae*, and *Shigella sonnei* when treated with methanol-extracted hemolymph compared to chloroform-extracted hemolymph. Differential responses of chloroform and methanol extracts of hemolymph support that the antibacterial activity is derived from small peptides, likely AMPs, but may also include lipid-derived antimicrobial activity (discussed in Lauric Acid section).

**Table 1.** Select reported microorganisms and corresponding antimicrobial peptide (AMP) activity from methanol-extracted hemolymph of the larvae of black soldier fly (*Hermetia illucens*)

Type	Bacteria	MIC^1^	Unit	Source
Gram-positive	Methicillin-resistant *Staphylococcus aureus* (MRSA)	25	mg/mL	Park et al., 2014
	*Kocuria rhizophila*	25	mg/mL	
	*Mircococcus luteus*	25	mg/mL	
	*Bacillus subtilis*	12.5	mg/mL	
	*Staphylococcus aureus*	100	mg/mL	
Gram-negative	*Enterobacter aerogenes*	25	mg/mL	
	*Pseudomonas aeruginosa*	12.5	mg/mL	
	*Escherichia coli*	12.5	mg/mL	
		4.67^2^	mm	Harlystiarini et al., 2019
		6.00^3^	mm	
	*Salmonella*spp.	4.33^2^	mm	
		6.33^3^	mm	
	*Helicobacter pylori*	8.00^4^	mm	Alvarez et al., 2019
	*Klesbiella pneumonia*	8.51	mm	Choi et al., 2012
	*Neisseria gonorrhoeae*	10.5	mm	
	*Shigella sonnei*	12.4	mm	
Yeast	*Candida albicans*	25	mg/mL	Park et al., 2014

MIC, minimum inhibitory concentration

160 mg/mL concentration of extract

320 mg/mL concentration of extract

Considered “most potent fraction”

While the hemolymph fraction appears to be optimal for detecting antimicrobial activity in vitro, research also shows that BSFL-derived ingredients, without further fractionation, exhibit similar activity. Additionally, when gastrointestinal digestion was simulated, activity was still retained. For example, BSFL protein meal digest significantly inhibited *C*. *perfringens* growth (Dong et al., 2021a), and digests of BSFL protein meal, chitin-rich BSFL protein meal, and mealworm larvae powder were tested against *C*. *difficile* and significantly reduced the damaging effect of toxin A on cell mucosal layers produced by *C. difficile* (Dong et al., 2021b). These data support that the ingredients being produced by commercial insect production systems have the potential to confer antimicrobial activity and optimize immune responses against typical challenges when fed as part of a complete nutritional program.

AMP activity has been demonstrated in vivo. In broiler chicks, incorporation of 1, 2, or 3% BSFL in the complete diet resulted in an increased number of CD4+ lymphocytes, increased lysozyme activity, and increased chick survival rates (67%, 75%, and 85%, respectively) against *Salmonella* Gallinarum compared to chicks fed no BSFL (50% survival) (Lee et al., 2018). Similarly, Beagle dogs supplemented with defatted BSFL meal (1 or 2%) for six weeks, then challenged with *E. coli* lipopolysaccharide had increased anti-inflammatory and antioxidative capacity compared to control dogs fed no BSFL meal (Lei et al., 2019).

BSFL may also modulate bacterial populations within their feed sources (Bessa et al., 2020), which has positive implications for the use of contaminated feed ingredients that would otherwise be slated for the landfill. For example, reduction of *Salmonella* spp. has been demonstrated by BSFL reared in cattle manure, chicken manure, and “fecal sludge” (Bessa et al., 2020). It is important to keep in mind that the expression of AMPs and the resulting efficacy are organism and diet specific, and may depend on additional factors, for example, age of larvae and rearing density (Kong et al., 2019; De Smet et al., 2021), as described below.

### Antimicrobial Peptide Characterization

Repeated and demonstrable antimicrobial activity from various BSFL fractions warrants further investigation into active components and specific mechanisms of activity. To date, there have been five categories of AMPs identified from BSFL hemolymph, including attacins, cecropins, defensins, diptericins, and knottin-like proteins, and three types of bacterial recognition proteins, including peptidoglycan recognition proteins (PGRPs), gram-negative bacteria binding proteins (GNBPs) and phenoloxidases (Vogel et al., 2018). All categories are described below except for GNBPs, which have not yet been directly correlated to known mammalian recognition proteins (Lee et al., 1996).

Attacins, a class of AMPs first reported in the giant silk moth (*Hyalophora cecropia*), have a random coil structure and are glycine-rich with half of the amino acids comprised of alanine, aspartic acid, phenylalanine, and threonine (Hultmark et al., 1983; Vilcinskas et al., 2013; Sheehan et al., 2018; Buonocore et al., 2021). The main mechanism of action is via alteration of the permeability of the cell membranes of gram-negative bacteria as attacins bind to lipopolysaccharides on the bacterial cell surface and block the translation of cell membrane proteins, allowing cecropins and lysozymes to enter the cell (Carlsson et al., 1991; Vilcinskas et al., 2013). Attacins have been identified in multiple insect species, with six genes coding for attacins in BSFL (Vogel et al., 2018), four genes in mealworm (adult and eggs; Jacobs et al., 2017), 10 genes in the adult harlequin lady beetle (*Harmonia axyridis*) (Vilcinskas et al., 2013), and three genes in the adult red flour beetles (*Tribolium castaneum*) (Vilcinskas et al., 2013).

Cecropins, the first identified insect AMP, also identified from the giant silk moth (*H*. *cecropia*), are α-helical linear antimicrobial peptides that lack a cysteine residue (Steiner et al., 1981; Brady et al., 2019; Buonocore et al., 2021). Their main mechanism of action is via cell membrane lysis resulting in cell membrane lesions in both gram-positive and gram-negative bacteria, although the response tends to be stronger against gram-negative bacteria (Andersons et al., 1991; Brady et al., 2019). Cecropins have been identified in mammals, specifically bovine adrenal glands and porcine intestines (Sheehan et al., 2018), as well as insects, including seven genes coding for cecropins have been identified in BSFL (Vogel et al., 2018), as well as four genes in *D*. *melanogaster* (Brady et al., 2019) and six genes in the black fly (*Simulium bannaense*) (Wu et al., 2015).

Defensin AMPs have been described in vertebrates, invertebrates, plants, and fungi. They are cysteine-rich cationic proteins predominantly expressed by epithelial cells or neutrophils (De Yang and Oppenheimer, 2003; Hiemstra, 2006; Vilcinskas et al., 2013; Machado and Ottolini, 2015). According to the vast array of literature regarding defensins, they have a broad range of purpose from antifungal effects (Thomma and Cammue, 2002; Sheehan et al., 2018) to mammalian sperm maturation, canine coat color expression, immune modulation via interaction with melanocortin receptors and wound healing (Machado and Ottolini, 2015). The main mechanism of action is similar to that of attacins, involving the alteration of bacterial (particularly gram-positive bacteria) membrane permeability (Machado and Ottolini, 2015; Sheehan et al., 2018), that induces membrane necrosis, affecting membrane electrostatic charge and resulting in the formation of membrane “pores” that allow other ions and nutrients to escape the cell (Machado and Ottolini, 2015). Vogel et al. (2018) identified 26 genes coding for defensin AMPs in BSFL, Jacobs et al. (2017) and Dubuffet et al. (2015) each identified one gene in adult *T*. *molitor* and the eggs, and Vilcinskas et al. (2013) identified 19 genes in adult *H*. *axyridis*. Additionally, Dubuffet et al. (2015) demonstrated that the defensin identified in *T*. *molitor* eggs was inherited from the maternal *T*. *molitor* as an evolved mechanism to protect offspring from infection.

Diptericins, another class of glycine-rich AMPs, first reported in the blowfly (*Phormia terranova*), demonstrate activity against gram-negative bacteria (Wicker et al., 1990; Reichhart et al., 1992; Kappler et al., 1993; Cudic et al., 1999). Diptericins were mostly researched in the latter decades of the 20th century. At that time, researchers differed in their conclusions as to the true classification of diptericins (some attributing diptericins to the attacin family), as well as lacking conclusions as to the mode of action of diptericins against bacteria (Wicker et al., 1990; Reichhart et al., 1992; Kappler et al., 1993; Cudic et al., 1999). Vogel et al. (2018) identified 10 genes coding for diptericin AMPs in BSFL.

Knottin-like proteins are families of disulfide-rich AMPs characterized by an inhibitor cysteine knot motif comprised of three interconnected disulfide bridges (Gracy et al., 2007; Postic et al., 2018). Knottin families come from a variety of species, including cone snails (conotoxins), spiders, and insects (Gracy et al., 2007). Knottins also have a variety of functions, including antimicrobial activity, due to cytotoxic/toxic activity and protease inhibition, and insecticidal activity (Gracy et al., 2007; Postic et al., 2018). They have high binding specificity for their target molecules, although the range of target molecules is very large due to the broad diversity of knottin proteins (Smith et al., 1998). Vogel et al. (2018) identified four genes coding for knottin-like AMPs in BSFL.

Peptidoglycan recognition proteins (PGRPs) are considered “pattern recognition receptors” that recognize and hydrolyze peptidoglycan within the wall of bacterial cells (Kang et al., 1998). Peptidoglycan triggers the prophenoloxidase cascade (Yoshida et al., 1996), a part of the innate immune response of insects (Kanost and Gorman, 2008; Lu et al., 2014). Thus, PGRPs play a critical role in the recognition of foreign cells by the insect immune system. Kang et al. (1998) isolated a PGRP from cabbage looper moth (*Trichoplusia ni*) larvae challenged with *Enterobacter cloacae*. The strongest PGRP expression was identified from the larval fat body, with some expression in hemocyte samples, and no expression in the gut or organs. This same PGRP was also isolated from mice and humans with demonstrated functional similarities (Kang et al., 1998). Because of the redundancy of expression of this molecule in vertebrates, it is possible that PGRPs derived from insects have efficacy in vertebrate immune recognition pathways.

Phenoloxidases are also part of the innate immune system in many invertebrate species, defending against gram-positive and gram-negative bacteria, as well as fungi and viral agents (Decker and Rimke, 1998). The defense is the result of tyrosinase hydroxylation, which leads to the oxidation of quinones, and the production of melanin around invading pathogens and wounds (Decker and Rimke, 1998; Sugumaran, 2002; Kanost and Gorman, 2008; Lu et al., 2014). The melanin itself can kill foreign parasites and pathogens (Kanost and Gorman, 2008).

The broad array of antimicrobial peptides expressed in various insect species, and demonstrated activity in vitro and in vivo, provides a unique opportunity to not only deliver essential nutrition to commercial livestock and companion animals but also the potential to improve markers of health and well-being by modulating either microbial communities or directly interacting with the vertebrate immune system. Further research is certainly needed to better characterize mechanisms, necessary concentrations for efficacy, and impacts of rearing and processing of insects on these components.

### AMP Activity Is Modified by Insect Rearing Conditions

The diet that is fed to insects has been shown to directly impact the expression of AMP genes. In BSFL, Vogel et al. (2018) demonstrated that AMP genes were differentially expressed when larvae were fed diets supplemented with either bacteria-enrichment (addition of *E*. *coli* BL21, *M*. *luteus*, *P*. *flourescens* BL915, and *B*. *subtilis*), brewer’s grains, cellulose, chitin, sulfonated lignin, or sunflower oil. All dietary treatments upregulated AMP gene expression relative to the control, but the largest numbers of significantly differentially expressed AMP genes resulted from larvae fed brewer’s grains diet or sunflower oil. The lowest number of expressed genes was from larvae fed the cellulose diet. The in vitro antimicrobial activity of hemolymph extracts (methanol- and chloroform-extractions) from insects fed these diets were tested and the strongest inhibitions of gram-negative bacteria (*E*. *coli* and *P*. *fluorescens*) were from larvae fed the brewer’s grains diet and the cellulose diet, while gram-positive bacteria (*M*. *luteus* and *B*. *subtilis*) were more widely inhibited by larvae fed the bacteria-enriched, cellulose, chitin, and sunflower oil diets. The inhibition assays performed by Vogel et al. (2018) indicate that the diet fed during the larval rearing stage can have a significant impact on the antimicrobial activities of the resulting larval extracts, and ingredient modulation was more effective than feeding live bacteria to BSFL.

While dietary addition of bacteria may not be as immunostimulatory as the addition of particular feed ingredients, direct injection with bacteria results in modulation of AMP expression. When challenged by *E*. *coli* inoculation, BSFL hemolymph demonstrated three to five times greater AMP activity against *H*. *pylori* than hemolymph from uninoculated BSFL (Alvarez et al., 2019). When BSFL were challenged with various bacteria (*E*. *faecalis*, *S*. *marcescens*, *B*. *animalis*, *B*. *breve*, *B*. *subtilis*) and hemolymph was extracted, the *B*. *subtilis*-challenged hemolymph had the greatest antimicrobial activity against both the tested gram-negative (*E*. *coli*) and gram-positive (*S*. *aureus*) bacteria (Park et al., 2016). Additionally, Park et al. (2015) inoculated BSFL with *S*. *aureus* and demonstrated greater AMP activity from corresponding hemolymph, isolated and identified specifically as a defensin, against MRSA and *E*. *coli* compared to hemolymph from uninoculated BSFL. In an inoculation challenge with *E*. *coli* or *M*. *luteus*, BSFL hemolymph indicated upregulation of AMP activity, specifically of phenoloxidases and lysozymes, compared to hemolymph from uninoculated BSFL (Zdybicka-Barabas et al., 2017). Upregulation of other identified AMPs has also been demonstrated, as *T*. *molitor* larvae hemolymph showed significantly increased attacin activity when inoculated with *S*. *aureus*, compared to uninoculated larvae (Dobson et al., 2012), and *M. separata* larvae had higher levels of attacins, cecropins and defensins when reared at higher larval density (Kong et al., 2019). Finally, environmental conditions can also impact AMP activity. Higher stocking density of armyworm (*Mythimna separata*) larvae resulted in significantly increased AMP activity against *B*. *subtilis*, *Edwardsiella ictaluri*, *S. aureus*, and *Vibrio anguillarum* (Kong et al., 2019). Together, these data demonstrate that antimicrobial activity can be modulated in response to diet, immune challenge (bacterial challenge in particular), and environmental/rearing conditions. This knowledge can be used by insect producers to enhance these value-added properties as appropriate for the animal species being fed with the insect-derived ingredients.

### Other Potential Immunomodulatory Factors

In addition to AMPs, BSFL is a source of additional compounds that may also modulate the immune responses of the animals to which they are fed, including lauric acid and chitin. Lauric acid is a saturated, medium-chain fatty acid with a 12-carbon backbone and is naturally found in high concentrations in oils such as those from coconut, palm, and BSFL (Dayrit, 2015). Compared to longer-chain fatty acids, lauric acid has increased energy potential in animal feeding systems due to the reduced need for lipolysis before absorption in the GI tract, and because enterocytes are able to use lauric acid directly as an energy source (Greenberger et al., 1965; Guillot et al., 1993). Lauric acid is generally not palatable in its purified form but readily accepted by animals in its natural form and thus can be used as a feed additive (Rabani et al., 2019). In addition to nutritional value, lauric acid has potent antibacterial and antiviral properties with the highest efficacy against gram-positive bacteria (Harlystiarini et al., 2019). Lauric acid inhibits biofilm formation, membrane biosynthesis (Kumar et al., 2020) and virulence factors (Ruzin and Novick, 2000), and may kill vegetative cells and spores (Schlievert et al., 2018; Yang et al., 2018).

It was demonstrated in vitro that *Campylobacter jejuni* is susceptible to lauric acid (Molatová et al., 2009), while in vivo, lauric acid from palm kernel fatty acid distillates fed at 5% inclusion, reduced *Campylobacter coli*, a food safety threat to the meat of broiler chickens (Zeiger et al., 2017). In swine, BSFL meal containing lauric acid yielded a half log reduction of *D-Streptococci* in weaned piglets when fed at 8% of the diet (Spranghers et al., 2018), and increased the number of *Lactobacillus* and *Bifidobacterium* while decreasing the total *E. coli* in weaning pigs at a 2% inclusion rate (Yu et al., 2020). In finishing pigs, BSFL meal significantly increased the abundance of *Lactobacillus, Pseudobutyrivibrio, Roseburia* and *Faecalibacterium* while decreasing the abundance of *Streptococcus* spp. (Yu et al., 2019). Siberian sturgeon fed 15% BSFL meal for 2 mo had improved intestinal morphology and gut microbiota composition (Józefiak et al., 2019; Yu et al., 2020). Complete replacement of soybean oil with BSFL oil at 5% inclusion in turkeys reduced the growth of *Enterobacteriaceae* and decreased IL-6 levels; 50% replacement lowered the TNF-α concentration (Sypniewski et al., 2020). These results were attributed to the high lauric acid content of BSFL.

Chitin is also of interest from BSFL and other insects and crustaceans. Chitin is the second-most abundant structural polymer in nature after cellulose, and an important part of the arthropod cuticle, accounting for up to 60% of insect dry weight, depending on species (Tellam et al., 2000; Merzendorfer, 2006; Doucet and Retnakaran, 2012). Chitin content of BSFL has been reported for larvae (3.6%), prepupae (3.1%), adults (2.9%), and sheddings left after pupae emergence (14.1%) (Wang et al., 2020). Chitosan, the fully deacetylated form of chitin obtained through further processing, is typically the focus of chitin research due to its high solubility that correlates to a broader range of applications and is reported to be nontoxic and biodegradable (Goosen, 1997; Bessa et al., 2020). It is important to note that true chitin content analysis is difficult. Acid detergent fiber (ADF) analysis has historically been used as an estimate of insect chitin and maybe most accurate when corrected for the presence of amino acids (Finke, 2007) or acid detergent lignin (Hahn et al., 2018). Chitin content and form varies with larval age, due to changes in total concentration and degree of crystallization (Wang et al., 2020). Thermal stability of chitin derived from larvae of different ages does not appear to vary (Wang et al., 2020). As mentioned, chitin may be isolated from crustaceans, including shrimp and crab, but insect chitin may be more available for functional use by the animal being fed. When treated with 6 N HCl, chitin isolated from both the silkworm (*Bombyx mori*) and “beetle larva cuticle” degraded more readily than chitin isolated from shrimp (Zhang et al., 2000). *N*-deacetylation was also easier with insect chitin than shrimp chitin, and chitinase affinity was higher for beetle larva chitin than shrimp chitin (Zhang et al., 2000).

The benefits of dietary insect chitin are still under review, but properties such as that of an antibacterial agent (Sudarshan et al., 1992; Shin et al., 2019), emulsifier (Hirsch et al., 2019), and prebiotic (Bruni et al., 2018) have been identified. Antibacterial activity has been demonstrated from chitosan isolated from *T*. *molitor,* which inhibited growth *of Bacillus cereus*, *Listeria monocytogenes*, *E*. *coli*, and *S*. *aureus* (Shin et al., 2019). Emulsifying properties have been demonstrated from chitin isolated from house cricket (*Acheta domesticus)* (Hirsch et al., 2019), and modulation of gut microbiome has been demonstrated in BSFL-meal fed rainbow trout (*Oncorhynchus mykiss*) as compared to fishmeal (Bruni et al., 2018). This result was attributed to chitin content of the BSFL meal diets (1.05 and 2.09%, as analyzed).

## CONCLUSIONS

Insect ingredient manufacturers have the opportunity to provide the animal feed and pet food industries with sustainable sources of high-quality nutrients and value-added components, and for this reason commercialization of insect rearing facilities for production of ingredients for animals (and humans) has expanded rapidly in the last decade. Increased value may be recognized from these ingredients, as they have demonstrated ability to modulate microbial communities in vitro and in vivo, and the potential to improve immune function in response to typical stressors. In particular, BSFL-derived ingredients offer not only AMP activity but lauric acid that may provide additive or synergistic efficacy. For all insect species being commercialized to date, chitin and chitosan also represent value-added components of the insect-derived ingredients. Further research is needed to better characterize functionality and in particular, the responsiveness of insects to their environment such that functionality may be optimized for a particular animal feeding application.
